# Hereditary Leiomyomatosis and Renal Cell Cancer in a Patient With Isolated Uterine Leiomyomas

**DOI:** 10.1097/og9.0000000000000058

**Published:** 2025-01-30

**Authors:** Shakiba Ardestani, Meghan McGrattan, Brittany Gillies, Raymond H. Kim, Mara Sobel

**Affiliations:** Department of Obstetrics and Gynecology, University of Toronto, the University Health Network, the Department of Genetics, Princess Margaret Cancer Centre, the Department of Genetics and the Department of Obstetrics and Gynecology, Mount Sinai Hospital, the Department of Genetics, Hospital for Sick Children, and the University of Toronto, Toronto, Ontario, Canada.

## Abstract

**BACKGROUND:**

Hereditary leiomyomatosis and renal cell carcinoma (HLRCC) is a rare autosomal dominant syndrome that is characterized by renal cell carcinoma, cutaneous leiomyomas, and uterine leiomyomas. Although HLRCC is usually identified in individuals with multiple cutaneous leiomyomas or a personal or family history of renal cell carcinoma, advances in histopathology have allowed detection of HLRCC in myomectomy and hysterectomy specimens.

**CASE:**

A 36-year-old patient with a large leiomyoma was referred to gynecology for heavy menstrual bleeding. After no response to medical management, she proceeded with elective myomectomy. Fumarate hydratase (FH) immunohistochemical staining of the myomectomy specimen and subsequent molecular genetic analysis of the *FH* gene confirmed a diagnosis of HLRCC.

**CONCLUSION:**

As more patients with HLRCC are identified with a primary presentation of leiomyomas, it is essential that gynecologists are familiar with this diagnosis. Health care professionals should inquire about skin lesions and family history of renal cell carcinoma in patients with symptomatic leiomyomas and refer patients with risk factors or pathology findings suggestive of HLRCC for genetic counseling.

## Abstract 2

As more patients with hereditary leiomyomatosis and renal cell carcinoma are identified with a primary presentation of leiomyomas, it is essential that gynecologists are familiar with this diagnosis.

**Teaching Points**

- Individuals with a high burden or early onset of leiomyomas should be screened for hereditary leiomyomatosis and renal cell carcinoma (HLRCC) (Box 1), and health care professionals should ensure that they inquire about skin lesions and family history of renal cell carcinoma. When HLRCC is suggested on pathology, molecular genetic analysis of the *fumurate hydratase (FH)* gene is necessary to confirm a diagnosis of HLRCC.
- When HLRCC is diagnosed, a multidisciplinary approach is needed. It is necessary to screen for renal cell carcinoma, and dermatologic examinations and gynecologic consultation should be considered. Early genetic counselling is reccomended, and patients seeking fertility may be offered preimplantation genetic testing.
- Hereditary leiomyomatosis and renal cell carcinoma is no longer thought to be strongly associated with development of leiomyosarcoma, and standard management of uterine leiomyomas is appropriate.

Hereditary leiomyomatosis and renal cell carcinoma (HLRCC) is a rare autosomal dominant syndrome (prevalence 1 in 200,000) characterized by renal cell carcinoma, multiple cutaneous leiomyomas, and uterine leiomyomas.^1,2^ Hereditary leiomyomatosis and renal cell carcinoma is caused by a germline pathogenic or likely pathogenic variant in the *fumarate hydratase* (*FH*) gene, which plays a role in the Krebs cycle; disruption of this pathway leads to tumorigenesis.^3^ Although HLRCC usually is identified in individuals with multiple cutaneous leiomyomas or a personal or family history of renal cell carcinoma, advances in histopathology have allowed detection of *FH* deficiency in myomectomy and hysterectomy specimens, providing a new opportunity to identify HLRCC and to screen for this highly aggressive form of renal cell carcinoma. Individuals with HLRCC have a high propensity for leiomyomas, with 80–90% of those affected developing symptomatic leiomyomas, which are larger, more numerous, and diagnosed earlier than in the general population.^1,4^

Although cutaneous leiomyomas, which are inflamed and exquisitely painful skin lesions, are pathognomonic for HLRCC, leiomyomas may be much more subtle in presentation. Many patients present with vague symptoms, such as back pain or pelvic pressure, or with gynecologic symptoms with a broad differential, such as heavy menstrual bleeding. Fewer cases of HLRCC-associated leiomyomas have been described in the absence of cutaneous leiomyomas, and given the prevalence of benign leiomyomas in the general population, the diagnosis may easily be missed.^4^ In addition to personal health surveillance and family screening, in a young, fertile population, the genetic implications of HLRCC necessitate thorough diagnostics and counseling to inform appropriate family planning. With this in mind, we review the case of a young patient with HLRCC in the absence of cutaneous leiomyomas who initially presented with abnormal uterine bleeding secondary to uterine leiomyomas.

## CASE

A 36-year-old patient, gravida 0, was referred with heavy menstrual bleeding. Initial imaging demonstrated a dominant stable 7-cm intramural leiomyoma with minimal impingement into the endometrial cavity. Her bleeding proved refractory to medical management, and she was actively trying to conceive. Therefore, she elected to proceed with myomectomy. She underwent an uncomplicated myomectomy, and her anatomy was thought to be broadly unremarkable. *FH* immunohistochemical staining of her surgical specimen subsequently identified a lack of *FH* protein expression within the myoma, indicating the possibility of an *FH* mutation. She was referred for genetic counseling and consented to molecular genetic analysis of the *FH* gene. She was found to harbor a likely pathogenic frameshift variant in the *FH* gene (NM_000143.3):c.734_735delGG (p.Gly245Alafs*4), confirming a diagnosis of HLRCC. Given her desire for pregnancy, she was referred to a fertility specialist to discuss the option of in vitro fertilization to allow preimplantation genetic testing of embryos for HLRCC. Her family was offered cascade genetic testing, and she initiated annual screening for renal cell carcinoma. Her partner was also offered germline *FH* genetic testing for assessment of reproductive risk for *FH* deficiency.

## DISCUSSION

Hereditary leiomyomatosis and renal cell carcinoma is a relatively new familial cancer syndrome, having been first described in 2001.^5^ Since then, affected families have been studied extensively, leading to the emergence of clinical criteria for screening. Therefore, HLRCC should be suspected and patients referred for genetic counseling when at least one major or two minor criteria are present (Box 1).^6^

Box 1. **Clinical Screening Criteria for Hereditary Leiomyomatosis and Renal Cell Carcinoma. Consider genetic referral with at least one major or two minor criteria.**

**Major criterion**
  The presence of multiple cutaneous leiomyomas
**Minor criteria**
  A solitary cutaneous leiomyoma and a family history of hereditary leiomyomatosis and renal cell carcinoma
  Surgical treatment for symptomatic leiomyomas before age 40 years
  Papillary type 2 renal cell carcinoma before age 40 years
  A 1st-degree relative who meets one of the aforementioned criteria

Cutaneous leiomyomas are typically the first presentation of HLRCC and appear at a mean age of 24 years. They are firm red or brown nodules most frequently seen on the torso, upper back, and upper and lower extremities. Pain is the hallmark feature, reported in approximately 90% of patients. Although the severity of pain is variable, approximately 20% of patients report moderate to severe impairment in quality of life.^1^ When cutaneous leiomyomas are suspected, a biopsy should be performed to confirm the diagnosis, followed by molecular genetic analysis of the *FH* gene.

Although cutaneous leiomyomas have previously been estimated to affect 71–100% of individuals with HLRCC, more recent studies have suggested that the overall prevalence may be closer to 48%, with an age-related prevalence to 75 years of 78%.^2^ It is therefore possible that many more patients than previously appreciated may present during their reproductive years with uterine leiomyomas in the absence of cutaneous lesions or without clinical features at all. Appropriate identification of patients at risk of HLRCC with the aforementioned screening criteria is therefore imperative, and health care professionals should ensure that they inquire about skin lesions and family history of renal cell carcinoma in patients presenting with symptomatic leiomyomas, particularly when they are young.

Leiomyomas are common benign smooth muscle tumors of the uterus. Although many leiomyomas are asymptomatic, large or multiple leiomyomas may cause heavy menstrual bleeding, bulk symptoms, infertility, and adverse obstetric outcomes. Many patients choose to undergo myomectomy or hysterectomy for the management of leiomyomas. Hereditary leiomyomatosis and renal cell carcinoma–associated uterine leiomyomas are often diagnosed earlier (mean age 28–30 years) than sporadic uterine leiomyomas and have a higher risk of surgical intervention and early hysterectomy.^7^ Previous publications have suggested an association between HLRCC-associated leiomyomas and leiomyosarcoma, a rare and aggressive smooth muscle cancer. Diagnosis of HLRCC-associated leiomyosarcoma was previously based on older diagnostic models. When these same samples were re-evaluated with updated World Health Organization diagnostic criteria for sarcomas, the vast majority of samples were reclassified as either benign mitotically active or atypical leiomyomas. The current opinion on this has therefore shifted, and leiomyosarcomas are thought to no longer, or only extremely rarely, be associated with HLRCC.^8^ Patients can therefore be reassured that there is no increased risk of leiomyosarcoma with HLRCC, and standard management of leiomyomas is appropriate.

In patients with leiomyomas in whom the possibility of HLRCC has been clinically overlooked, the first suspicion of a genetic syndrome may come from their surgical pathology after myomectomy or hysterectomy. Immunohistochemistry can now detect *FH* protein deficiency in leiomyoma tissue, which represents either a germline mutation associated with HLRCC or a somatic mutation within the leiomyoma. Formal genetic testing is recommended in patients with *FH*-deficient leiomyomas to identify HLRCC cases.

When HLRCC is identified, a multidisciplinary approach is necessary to address the multiple facets of this syndrome. Hereditary leiomyomatosis and renal cell carcinoma carries an estimated 15–20% lifetime risk of renal cell carcinoma, which can develop at an early age and is often more aggressive (particularly papillary renal cell carcinoma) than non-HLRCC renal cell carcinoma.^9,10^ Although no definitive surveillance guidelines are established, contrast-enhanced magnetic resonance imaging every 6–12 months is recommended for patients with known HLRCC. For family members of those with HLRCC, genetic testing for late-presenting genetic disorders is typically recommended by age 16–18 years or when the patient can appropriately consent; however, given the risk of renal cell carcinoma in very young patients, testing as early as age 8–10 years may be considered.^11^ In patients with HLRCC, some authors suggest dermatologic examination every 1–2 years for evidence of cutaneous leiomyomas,^12^ and although there are no firm gynecologic guidelines, referral to a gynecologist at the onset of heavy menses or other symptoms of leiomyomas would be prudent. For patients wishing to conceive, preimplantation genetic testing for monogenic disorders may be offered in which embryos created during in vitro fertilization are tested before implantation in the uterus, allowing parents to elect to implant only unaffected embryos.^12^ In addition, given the risk of autosomal recessive *FH* deficiency caused by biallelic germline *FH* pathogenic variants, fumarate hydratase germline genetic testing can also be offered to reproductive partners for family planning purposes. Thus, emphasis should be placed on early detection, symptom management, and appropriate family planning counseling.
